# Selection of Lactic Acid Bacteria Isolated from Fresh Fruits and Vegetables Based on Their Antimicrobial and Enzymatic Activities

**DOI:** 10.3390/foods9101399

**Published:** 2020-10-02

**Authors:** José Rafael Linares-Morales, Guillermo Eduardo Cuellar-Nevárez, Blanca Estela Rivera-Chavira, Néstor Gutiérrez-Méndez, Samuel Bernardo Pérez-Vega, Guadalupe Virginia Nevárez-Moorillón

**Affiliations:** Facultad de Ciencias Químicas, Universidad Autónoma de Chihuahua, Circuito Universitario s/n, Campus II, Chihuahua 31125, Mexico; joselinares2003@yahoo.com (J.R.L.-M.); gcuellar_nevarez@hotmail.com (G.E.C.-N.); bchavira@uach.mx (B.E.R.-C.); ngutierrez@uach.mx (N.G.-M.); sperez@uach.mx (S.B.P.-V.)

**Keywords:** antimicrobial, enterococcus, enzymes, exopolysaccharides, food safety, Leuconostoc, vegetables

## Abstract

Lactic acid bacteria (LAB) are an important source of bioactive metabolites and enzymes. LAB isolates from fresh vegetable sources were evaluated to determine their antimicrobial, enzymatic, and adhesion activities. A saline solution from the rinse of each sample was inoculated in De Man, Rogosa and Sharpe Agar (MRS Agar) for isolates recovery. Antimicrobial activity of cell-free supernatants from presumptive LAB isolates was evaluated by microtitration against Gram-positive, Gram-negative, LAB, mold, and yeast strains. Protease, lipase, amylase, citrate metabolism and adhesion activities were also evaluated. Data were grouped using cluster analysis, with 85% of similarity. A total of 76 LAB isolates were recovered, and 13 clusters were formed based on growth inhibition of the tested microorganisms. One cluster had antimicrobial activity against Gram-positive bacteria, molds and yeasts. Several LAB strains, PIM4, ELO8, PIM5 and CAL14 strongly inhibited the growth of *L. monocytogenes* and JAV15 and TOV9 strongly inhibited the growth of *F. oxysporum*. Based on enzymatic activities, 5 clusters were formed. Seven isolates hydrolyzed starch, 46 proteins, 14 lipids, and 36 metabolized citrate. LAB isolates with the best activities were molecularly identified as *Leuconostoc mesenteroides*, *Enterococcus mundtii* and *Enterococcus faecium*. Overall, LAB isolated from vegetables showed potential technological applications and should be further evaluated.

## 1. Introduction

Despite the vast variety of lactic acid bacteria (LAB), only a small percentage are technologically employed in food production. LAB are commonly isolated from dairy products; however, fresh vegetable sources have gained importance, since they are also an inexhaustible source of LAB. The isolates from fresh vegetables can be novel LAB strains with enhanced characteristics, or their metabolites could be useful in food production. The diversity in plant ecosystems provides a challenge for the LAB strains to adapt to vegetable and fruit environments, and these capabilities vary significantly among species and strains [1]. Therefore, a considerable diversity of LAB can be found in fresh vegetables. *Lactobacillus plantarum* [2,3,4,5] currently referred to as *Lactiplantibacillus plantarum* comb. nov. [6], *Leuconostoc pseudomesenteroides* [3,7], *Weissella confusa* [3,4] and *Leuconostoc mesenteroides* [3,8] are counted among the LAB obtained from fresh fruits and vegetables.

The evaluation of LAB as biocontrol agents has gained attention [2,5,9] becoming a sustainable solution for food protection. Siroli et al. [5] recommended the use of biocontrol agents obtained from the same environment where they will be applied, since these investigators determined antimicrobial activity against pathogens inoculated and spoilage microorganisms naturally present on apple and lettuce. Di Cagno et al. [10] suggested screening starter cultures among the autochthonous microbiota of vegetables and fruits due to the high probability that these strains will guarantee extended shelf life while keeping the desired nutritional, rheological, and sensory characteristics. Furthermore, novel strains of LAB have been evaluated as fermentation starters [9,11,12,13,14]. In the preparation of fermented foods, it is expected that the addition of chemical products to avoid the growth of unwanted microorganisms; however, the use of those compounds can affect the growth of starter cultures. Consequently, the application of LAB with antimicrobial capacity is deemed a harmless approach to maintain safe food processing [15].

LAB can also be a source of novel strains with enzymatic activities of interest for food production or other industrial use of enzymes [16,17]. Enzymes of microbial origin are more desirable than enzymes from other sources as they may show enhanced catalytic activities, present higher yields, and economic culture media can be used in microbial growth. The primary enzymes employed for food production include amylases, proteases, and lipases [17]. Amylase [18], citrate lyase [19], and protease [11,14] activities have been previously reported in LAB isolated from vegetable sources. Another useful characteristic of LAB is the production of exopolysaccharides, which are able to enhance organoleptic characteristics and stability of food [16] and can enhance the adhesion and colonization of probiotic bacteria to the gastrointestinal tract [20].

There are many strains of LAB with technological potential yet to be discovered. This research was directed to assess the antimicrobial, enzymatic, and adhesion activities of LAB strains isolated from vegetable sources in Chihuahua, Chih., Mexico, to determine their potential for technological applications as alternative sources for the food industry.

## 2. Materials and Methods 

### 2.1. Isolation of Lactic Acid Bacteria

LAB were isolated from vegetable samples of chilaca chili, guava, green apple, jalapeño chili, corn, orange, zucchini, peach, red apple, pear, green tomato, pomegranate, lettuce, mandarin, cucumber, bell pepper, grape, soybean sprout, and nopal flower. A total of 153 samples of fruits and 21 samples of vegetables, unprocessed were obtained from Farmlands, Street markets, and Supermarkets in Chihuahua City, Mexico. The samples were placed in sterile bags and transported to the laboratory. A total of 10 mL of maximum recovery diluent (MRD; 8.5 g/L NaCl, 1 g/L peptone) were added to wash the surfaces of the samples. A total of 1 mL of MRD was transferred to tubes with 5 mL of De Man, Rogosa, and Sharpe (MRS) broth (Difco, Franklin Lakes, NJ, USA) and incubated at 26 °C from 18 to 24 h in an anaerostat. Later, a sample loop from each tube with growth was streaked on MRS agar (Difco, Franklin Lakes, NJ, USA) and incubated under the same conditions mentioned before. LAB characteristic colonies (white, opaque, small and convex), Gram-positive, catalase-negative characterized through traditional biochemical tests [21] were streaked again on MRS agar and incubated at 26 °C for 18 h. Pure cultures were grown in MRS broth under the same conditions and transferred to sterile glycerol to a final concentration of 40% and maintained as stock cultures at −20 °C. 

### 2.2. Antimicrobial Activity of LAB Cell-Free Supernatants

The antimicrobial activity of LAB cell-free supernatant (LAB-CFS) was determined as the percentage of inhibition of indicator microorganisms when were incubated in a medium supplemented with a LAB-CFS. This assessment was carried out following the microtitration method reported by Inglin et al. [22] with variations. A total of 100 µL of Brain Heart Infusion (BHI) broth for bacteria (Bioxon, Cuautitlán Ixcalli, México) or YM broth (yeast extract 3 g/L, malt extract 3 g/L, dextrose 10 g/L, Peptone 5 g/L) for yeast and molds were poured into a 96 well microplate. Both media were previously supplemented with K_2_HPO_4_ (0.1 M) to buffer the lactic acid present in the LAB-CFS, and discriminate against the low pH antimicrobial effect. Additionally, 30 µL of microbial suspensions of the indicator microorganisms (Table 1) were added to the wells. The suspension was adjusted to 1.5 × 10^8^ cel/mL for bacteria and yeast (using the McFarland 0.5 standard) and 1 × 10^3^–1 × 10^4^ mold spores (adjusted by counting the spores with a New Bauer chamber). Finally, 50 µL of centrifuged (3800× *g*, 25 min; Eppendorf 5810R, Hamburg, Germany) and pasteurized (75 °C, 3 min) LAB-CFS were also placed in the wells of the same 96 well microplate by triplicate. Negative controls were set in one row with 150 µL of BHI broth or YM broth supplemented with K_2_HPO_4_ (0.1 M) and suspensions of each indicator microorganisms. Optical density (OD_0_) was measured at 595 nm, using a Microplate reader (Biorad 550, Japan). Microplates were incubated under conditions suitable for each indicator microorganism (Table 1). Optical density was measured again at the end of incubation (OD_f_). Antimicrobial activities (AA) were calculated according to Wong et al. [23] as follows:

AA = 100 − ((ΔDOs/ΔDOc) ∗ 100)(1)

where ΔDOs is the average of the difference between initial (OD_0_) and final (OD_f_) optical density of the samples, and ΔDOc is the average of the difference (OD_0_ and OD_f_) of the optical density of the negative control. AA values higher than 50% were considered as strong inhibition, values between 15 and 49.99% were deemed to be moderate inhibition, values lower than 14.99% were considered as non-significant inhibition. 

### 2.3. Enzymatic Capacity

For the evaluation of the enzymatic capacity of the LAB isolated from vegetable sources, three culture media were prepared. Skim milk agar [24] for protease (skim milk 1.5% and agar 1.5%), tributyrin agar for lipase (MRS agar, tributyrin 1%), starch agar for amylase activity (M17 agar; starch 1%) flooded with Gram’s iodine solution after incubation [25]. Every plate media was puncture inoculated with an overnight LAB culture and incubated at 26 °C for 24–48 h. The presence of a halo around the puncture was considered a positive activity. Citrate metabolism of LAB was assessed as described by Kempler and McKay, [26] employing citrate agar (1% milk powder, 0.25% casein peptone, 0.5% glucose, and 1.5% agar), complemented with a 10 mL potassium ferricyanide solution (potassium ferricyanide 10%, iron citrate 2.5% and sodium citrate 2.5%). Citrate agar plates were also puncture inoculated with an overnight LAB culture and incubated at 26 °C for 24 h. Blue colonies were considered positive for citrate metabolism. The enzymatic activities were reported in a semi-quantitative scale of a negative result (−) or different intensities of positive results (+, ++, +++), determined by visual comparison of results among the different isolates.

### 2.4. Biofilm Formation 

To evaluate the production of exopolysaccharides by LAB, a biofilm formation assay was done according to the method reported by Stepanović et al. [27] with modifications. LAB strains were inoculated in tubes with MRS broth supplemented with 2% sucrose. From each tube, 200 µL were placed in a 96 wells microplate by triplicate. As a negative control, 200 µL of MRS broth were placed in triplicate. The microplate was incubated in an anaerostat at 26 °C for 48 h. After incubation and staining of the EPS adhered to the microplate well with crystal violet, rinsing with tap water, followed by solubilization with acetic acid, the optical density (595 nm) was measured using a microplate reader Biorad 550 (Hercules, CA, USA). All tests were carried out by triplicate, and the results were averaged. The adhesion capacity was assessed according to the scale proposed by Stepanović et al. [27].

### 2.5. Molecular Identification of LAB

#### 2.5.1. DNA Extraction 

The DNA extraction was carried out according to Pospiech and Neumann [28] with variations. A sample loop of each LAB was added to a 1.5 mL Eppendorf tube with 400 μL of TE 1X Buffer (10 mM Tris-HCl, one mM Na_2_EDTA, pH 8.0) and 50 μL of Lysozyme (10 mg/mL). The mix was incubated for 1.5 h at 37 °C. Then, 70 μL of SDS (10%) and five μL of Proteinase K (10 mg/mL) were added and the mix was homogenized in a vortex and incubated at 65 °C for 10 min. Later, 100 μL of 5 M NaCl and 100 μL of a solution of Cetyltrimethylammonium bromide (CTAB) and NaCl (CTAB 100 mg/mL, NaCl 41 mg/mL) were added, the mix was homogenized again in a vortex and incubated for 10 min at 65 °C. Then, 700 μL of chloroform/Isoamyl alcohol (24:1) were added, the mix was homogenized in a vortex and centrifuged at 10,000× *g* for 10 min and 20 °C. The supernatant was transferred to a new tube, and 0.6 volumes of Isopropyl alcohol were added. The DNA was left to precipitate at −20 °C for 24 h. The DNA was recovered by centrifugation at 10,000× *g* and 20 °C for 15 min and decant the supernatant. The DNA pellet was washed twice with 600 μL of 70% ethyl alcohol and subsequent centrifugation at 10,000× *g* and 4 °C for 5 min and decant the supernatant. Finally, the pellet was dried at room temperature, suspended in 50 μL of Mili-Q water and maintained at −20 °C. 

#### 2.5.2. Amplification of the 16s rDNA

Amplification of the 16s rDNA coding region of each LAB was performed by polymerase chain reaction (PCR), according to Liu et al. [29] with modifications. A reaction mixture with a final volume of 25 μL was used. The mixture contained 5 μL of each LAB DNA (100 ng), 2.5 μL of Mili-Q water, 12.5 μL of Go Taq^®^ Colorless Master Mix 2X (Promega Corporation, Madison, WI, USA; GoTaq^®^DNA Polymerase 2X; colorless reaction Buffer pH 8.5; 400 µM dATP, 400 µM dGTP; 400 µM dCTP; 400 µM dTTP and 3 mM MgCl2.) and 2.5 μL of each primer (10 μM). The primers used were 27F- 5′ AGA GTT TGA TCC TGG CTC AG 3′, and 1492R- 5′ TAC GGT TAC CTT GTT ACG ACT T 3′(Integrated DNA Technologies, Coralville, IA, USA), according to Liu et al. [29]. The reaction was carried out in a Thermocycler (Biorad, T100 Thermal cycler,). The amplification program was as follows: pre-denaturation at 95 °C for 10 min, 30 cycles consisting of denaturation at 93 °C for 1 min, annealing at 50 °C for 1 min and extension at 72 °C for 1.5 min. Finally, an ultimate extension at 72 °C for 5 min was carried out. The PCR products were purified with Amicon columns and confirmed by using agarose 2% (*w*/*v*) gel electrophoresis stained with Syber Safe DNA gel stain (Invitrogen), a Low DNA mass ladder (Invitrogen) in 1X Tris Borate EDTA (TBE 1X) buffer at 90 V, for 40 min. The images were obtained with a Kodak Gel Logic 2200 Imaging System (Eastman Kodak Company), and DNA was quantified from the gels.

#### 2.5.3. Sequencing

The amplified samples of LAB 16s rRNA gene were sent to Macrogen Corp. (Rockville, MD, USA) to be sequenced by the Sanger method with the primers described for the amplification. The LAB 16s rRNA sequences (Forward and Reverse) obtained were pre-analyzed, comparing them with the sequences gathered in the National Center for Biotechnology Information (NCBI) GenBank database through the Standard nucleotide Basic Local Alignment Search Tool program (https://blast.ncbi.nlm.nih.gov/Blast.cgi) to determine whether both sequences matched with the same microorganism sequence. Later, in order to improve the percentage of similarity, a reverse complement sequence was generated using the bioinformatics.org online tool. Subsequently, the sequences (Forward and Reverse complement) were aligned using the SeaView 3.2 (http://134.214.32.76/software/seaview3.html), and the resulting sequences were analyzed with the Standard nucleotide Basic Local Alignment Search Tool program from the NCBI GeneBank database. Finally, the sequences were submitted to the GeneBank submission portal to obtain accession numbers. Nucleotide sequences were also analyzed with the Clustal.X software (http://clustal.org/clustal2/) and Mega X software (https://www.megasoftware.net/) to generate a phylogenetic tree.

### 2.6. Statistical Analysis 

To select the best candidates for antimicrobial and enzymatic activities among the LAB isolates, the results of inhibition against all indicator microorganisms (AA percentages of LAB-CFS), as well as the result of enzymatic activities were analyzed. A hierarchical clustering analysis using a Euclidean distance metric with complete linkage, with 85% of similarity was used to determine the membership of each isolate to the different clusters. A dendrogram for the antimicrobial and enzymatic activities were generated from the analysis. The statistical analysis was done using the Minitab 18 software (Minitab, Inc., State College, PA, USA).

## 3. Results

### 3.1. Lactic Acid Bacteria Isolation

From a wide variety of vegetables, a total of 76 presumptive LAB isolates were obtained and screened for antimicrobial and enzymatic capacities. Table 2 shows the fruit and vegetable sources of LAB. The highest amounts of LAB were isolated from chilaca chilis (10), guava (8), green apples, jalapeño chilis, corn (6), and oranges (5). LAB were also isolated from zucchini, peaches, red apples, pears, and green tomatoes (4). LAB were less commonly isolated from pomegranates, lettuce, mandarin, cucumbers, bell peppers, grapes, soybeans sprouts, and nopal flowers.

### 3.2. Antimicrobial Activity of LAB Cell-Free Supernatants

**Figure 1:**
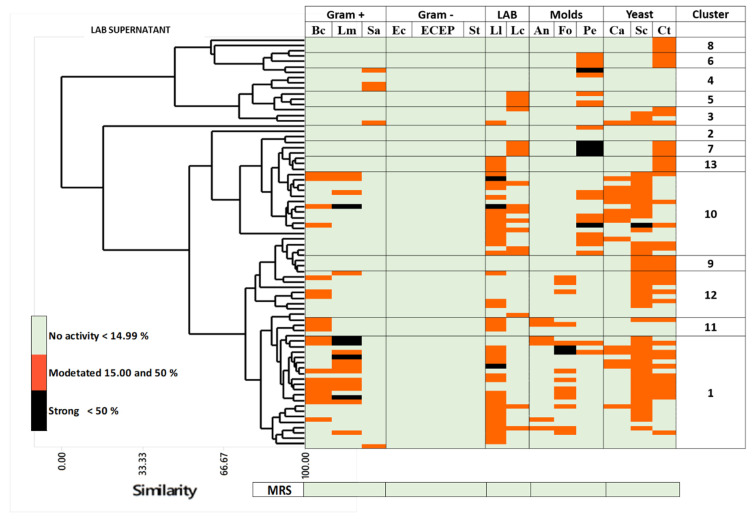
Dendrogram based on the Antimicrobial activity percentages of Lacti Acid Bacteria cell-free supernatants (LAB-CFS) and sterile De Man, Rogosa, and Sharpe (MRS) broth against indicator microorganisms. B.c.: *B. cereus*, L. m.: *L. monocytogenes*, S. a.: *S. aureus*, E. c.: *E. coli*, ECEP: *E. coli* O157:H7, S. t.: *S.* Typhimurium, L. l.: *L. lactis*, L. c.: *Lacticaseibacillus casei*, A. n.: *A. niger*, F. o.: *F. oxysporum*, P. e.: *P. expansum*, C. a.: *C. albicans*, S. c.: *S. cerevisiae*, C. t.: *C. tropicalis*.

A dendrogram (Figure 1) was built based on the percentages of AA obtained from indicator microorganisms when they were incubated with the LAB-CFS. Through a hierarchical clustering analysis using 85% similarity, 13 clusters were formed. The AA of sterile MRS broth against indicator microorganisms was also included in Figure 1. LAB-CFS had antimicrobial activity mostly against Gram-positive bacteria (*B. cereus*, *L. monocytogenes*), LAB (*L. lactis*, *L. casei*), molds (*A. niger*, *F. oxysporum*, *P. expansum*) and yeast (*S. cerevisiae*, *C. albicans*, *C. tropicalis*). There was no growth inhibition against Gram-negative bacteria.

Cluster one gathered those LAB-CFS whose AA varied mainly from moderate to strong inhibition in all the microbial indicator groups except the Gram-negative bacteria. The seven strains from cluster one whose cell-free supernatants presented strong inhibition against some indicator microorganisms were selected for molecular identification. These strains were ELO8, PIM5, CAL14, and PIM4, which strongly inhibited the growth of *L. monocytogenes*. JAV15 and PEP11 strongly inhibited the growth of *L. lactis* and JAV15 and TOV9 strongly inhibited the growth of *F. oxysporum*.

### 3.3. Enzymatic and Adhesion Capacity

Table 3 shows the enzymatic and adhesion capacity of LAB isolated from fresh vegetable sources. For protease activity, 46 (60.5%) of the total of LAB isolates (76) obtained from vegetable sources were positive, only 14 isolates (18.42%) presented lipase activity, 36 isolates (47.37%) showed the ability to metabolize citrate, and only 5 (6.58%) were amylase positive. Furthermore, less than half of the isolates (35) had no adhesion activity, while more than half of the strains (41) displayed adhesion capacity from medium to high. 

**Figure 2:**
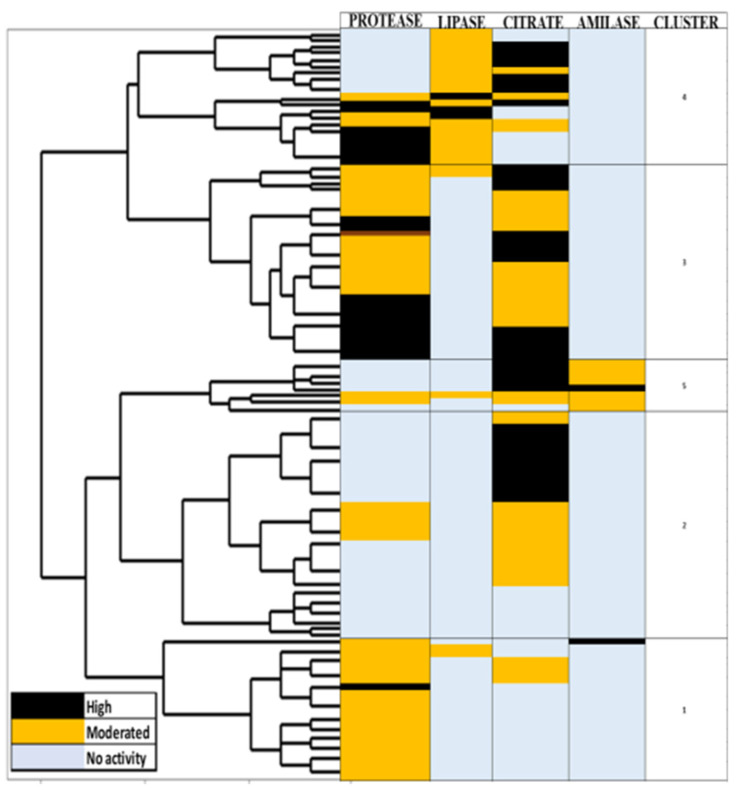
Dendogram of the behavior of LAB enzymatic activity (protease, lipase, amylase, citrate metabolism) grouped in clusters with a similarity of 85%.

Figure 2 describes the behavior of LAB isolates enzymatic activities grouped in eight clusters using an 85% similarity. LAB isolates with three or four enzymatic activities were selected for molecular identification. Cluster one included an isolate (NAR1) that was able to degrade proteins, citrate, and starch and also exhibited a strong adhesion capacity (Table 4). Two isolates in cluster three, ELO8 and PEP12, obtained from corn and cucumber, respectively, were positive for protease, lipase, and citrate metabolism and presented moderate adhesion capacity (Table 4). There were four LAB strains in cluster four obtained from guava (GUA13), green jalapeño (JAV15), red apple (MAR15), and bell pepper (PIM4), which presented protease and lipase activity, as well as citrate metabolism activities from moderate to high. GUA13 and PIM4 exhibited moderate adhesion activity (Table 4), while JAV15 and MAR15 did not present any adhesion activity (Table 4). Furthermore, an isolate obtained from tangerine (MAD3) expressed moderately the four enzymatic capacities evaluated and displayed a strong adhesion capacity.

### 3.4. Molecular Identification

**Figure 3:**
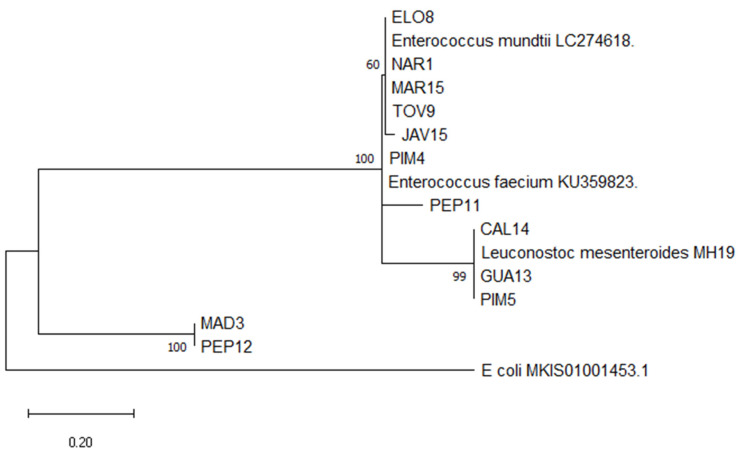
Phylogenetic tree of LAB isolated from fresh vegetables. The 16s rDNA gene sequences were aligned and the evolutionary distribution was inferred by applying the Maximum Likelihood method. The tree with the highest log likelihood (−1775.71) is presented. Initial tree(s) for the heuristic search were obtained automatically by using Neighbor-Join and BioNJ algorithms to a matrix of pairwise distances estimated using the Maximum Composite Likelihood (MCL) approach, and then selecting the topology with superior log likelihood value. The tree is drawn to scale, with branch lengths measured in the number of substitutions per site. This analysis involved 16 nucleotide sequences. There was a total of 366 positions in the final dataset. Evolutionary analyses were carried out in MEGA X software.

A description of selected LAB identification is presented in Table 5. According to the 16s rDNA gene sequence comparison, five of the isolates selected for their antimicrobial, enzymatic, and adhesion capabilities were identified as *L. mesenteroides*, five were identified as *E. mundtii* and two were *E. faecium* (Figure 3).

## 4. Discussion 

Although the search for LAB strains with biotechnological potential has been reported before, the search for new candidates that can be used as bioprotective agents in food processing is still much needed. This work is aimed to collect presumptive LAB isolates from fresh vegetables in the area of Chihuahua City, Mexico, and to characterize them in relation to metabolic traits that can be useful for the food industry. This works was aimed to search for new LAB isolates with possible biotechnological potential and was not directed to obtain a microbiome map of LAB populations in the fruits and vegetables analyzed. LAB have been commonly isolated from fresh vegetables, as shown in diverse publications [2,3,4,7]. Moreover, many authors have reported the antimicrobial activity of LAB isolated from fresh vegetables against bacteria, yeast, and molds [2,4,5,6,7,12,30].

Among the properties sought in LAB used for technological applications are pH reduction and antimicrobial metabolites [31]. LAB isolated from fresh vegetable sources in this research showed antimicrobial activity against Gram-positive pathogens (*B. cereus*, *L. monocytogenes*), LAB (*L. lactis*, *L. casei)*, molds (*F. oxysporum*, *P. expansum*), and yeast (*C. albicans*, *S. cerevisiae*, *C. tropicalis*). One of the main products of LAB fermentation, are organic acids mainly lactic acid, and the consequent low pH is in part, responsible for the antimicrobial activity of LAB-CFS. Commonly, the LAB supernatant is neutralized before further antimicrobial analysis, particularly for bacteriocin analysis. The methodology we used include the use of a buffered broth in the microtiter inhibition, by adding of potassium diphosphate (K_2_HPO_4_) [22,23]. The buffer capacity of the BHI and YM broth added with potassium diphosphate was tested by adding the LAB-CFS, with a pH above 6.0 in all cases (data not shown).

Based on the cluster analysis, isolates form Cluster 1 were selected for molecular identification. According to the 16s rDNA gene sequence comparison, two isolates selected due to their antimicrobial activity (PIM5, CAL14) were identified as *L. mesenteroides*, three more (ELO8, JAV15, TOV9) were *E. mundtii,* and two (PEP11, PIM4) were identified as *E. faecium*. *Leuconostoc* and *Enterococcus* are among the LAB genera most frequently isolated from raw fruits and vegetables [32]. The most frequently isolated LAB genus found in plants is *Leuconostoc,* and *L. mesenteroides* is the main specie associated with fresh vegetables [23] including Curly kale [20], cocoa beans [19] and Faba beans [14]. Furthermore, *Enterococci* as *E. mundtii* and *E. faecium* are commonly isolated from plant material [32] like fresh fruits and vegetables [33], cereals [13,30], chickpea sourdough [11], and fermented kidney beans flour [15] among others. Among the *E. mundtii* isolates identified, TOV9 showed antimicrobial activity against seven of the indicator microorganisms (*L. monocytogenes, L. lactis*, *F. oxysporum*, *P. expansum*, *C. albicans*, *S. cerevisiae*, and *C. tropicalis*). Antimicrobial activity against *L. monocytogenes* by *E. mundtii* isolated from vegetables has been previously reported [12,34].

Among the *L. mesenteroides* identified, CAL14 had the best antimicrobial profile since it inhibited the growth of *B. cereus*, *L. monocytogenes* (Strongly), *L. lactis*, *F. oxysporum*, *S. cerevisiae*, and *C. tropicalis*. It is also common to isolate strains of *L. mesenteroides*, which is capable of growth inhibition of *L. monocytogenes* [5,12,14,34] or *B. cereus* [35]. *E. mundtii* and *L. mesenteroides* isolates from the present report did not inhibit the growth of *S. aureus*, *E. coli*, or *S*. Typhimurium even though this activity has been widely reported [5,12,29,34,35]. Among the *E. faecium* identified, PIM4 showed antimicrobial activity against *B. cereus*, *L. monocytogenes* (Strongly), the three molds evaluated, as well as *S. cerevisiae*, and *C. tropicalis*. *E*. *faecium* strains have been reported with antimicrobial activity against *B*. *cereus* and *E*. *coli* [36] and *L*. *monocytogenes* and *E*. *coli* [37]. However, *E*. *faecium* strains in this research did not inhibit the growth of *E*. *coli*. Although the results are promising for the use of LAB isolates for post-harvest treatments, the safety of the strains need to be assessed. There are concerns of *Enterococcus* strains, since some can harbor pathogenic genes [37]; therefore, before considering the use of the isolates presented here, further studies are needed on the presence and expression of pathogenic traits.

The methodology used for the screening of antimicrobial activities of LAB isolates has been reported before, being its grater advantage, the screening of a large number of isolates, and indicator microorganisms [22,23]. The microtitration method has also been compared in effectiveness with the agar diffusion agar test, and it was observed that when OD inhibition was larger than 70%, correlated with a positive test in the agar diffusion test [37]. In the case of the strains reported here, inhibition was lower than 65%, and in the agar diffusion test, there was no clear zone of inhibition, although a decrease in the density of the bacterial lawn was observed. 

Vegetable samples collected were a good source of presumptive LAB isolates with important enzymatic activities, which can influence a food product with enhanced flavor, texture, or nutritional features. Additionally, these enzymatic properties can be used in the production of substances with industrial importance, such as lactic acid and diacetyl [18,38] or for the production of enzymes to be used at an industrial scale, such as amylase [17,18]. The LAB isolates selected bases on their enzymatic activity were also identified by molecular techniques. Four strains were identified as *E. mundtii* (ELO8, JAV15, MAR15, and NAR1), three as *L. mesenteroides* (MAD3, PEP12, and GUA13), and one as *E. faecium* (PIM4).

It is expected that LAB isolated from a proteinaceous substrate such as kidney beans produce proteases, as reported before [15]. Similarly, LAB isolated from a non-proteinaceous substrate like olive oil brines, do not expressed protease activity [31]. Although LAB isolates in this research were obtained from non-proteinaceous substrates, more than half displayed protease activity, showing potential for application in the manufacture of fermented dairy and meat products. All the LAB molecularly identified (*E. mundtii*, *E. faecalis*, and *L. mesenteroides*) were positive for proteases; isolation of proteolytic strains of *E. faecalis*, *E. faecium*, and *L. mesenteroides* from non-proteinaceous vegetable substrates have also been reported before [30,35,39]. Protease activity is a significant feature for technological applications since proteolytic strains can increase sensorial food attributes, including the increase of free essential amino acids or flavor enhancement. Furthermore, the digestibility of polypeptides is improved, and bioactive peptides with useful activities can be released [14,15]. Furthermore, LAB have been reported as immunomodulatory probiotics reducing the hypersensitivity to cow’s milk proteins in children [40]. 

Less than a quarter of the LAB isolates presented lipolytic activity. Nevertheless, a moderate or a lack of lipolytic activity has been reported for LAB isolates before [39,41,42]. Lipolytic activity is an advantageous characteristic of LAB for cheese ripening due to the breakdown of triglycerides into simpler molecules like fatty acids or mono and diglycerides [32,43]. Furthermore, it can synthesize esters at the later step of ripening when a_w_ is low [43]. Products of lipolytic activity on milk fat positively modify the aroma of dairy products, conferring the characteristic flavors of many cheese varieties [39]. 

Almost half of the LAB isolates metabolized citrate. Furthermore, all the strains chosen for molecular identification metabolized citrate. LAB can produce creamy and buttery flavor notes due to their capacity to metabolize citrate and transform it into diacetyl and acetoin. This characteristic aroma is a significant sensorial feature of dairy products [33,44] chocolate [19], and even sourdough bread [38]. Strains of *L. mesenteroides* are diacetyl [8,45] and acetoin producers [19]. *E. faecium* strains are also considered helpful in cheese manufacturing due to the production of desirable flavors associated with citrate metabolism [45]. Proteolytic and lipolytic activities, production of acetaldehyde, acetoin, and diacetyl, are important for cheese ripening [32,33]; therefore, *L. mesenteroides* is considered one of the most valuable LAB from an economic standpoint [33]. Although *E. faecium* is recognized as a pathogenic microorganism, it has been demonstrated its participation in the production of metabolites that add particular flavors to different cheese types [45].

LAB are not commonly associated with amylolytic activity even though some LAB with this characteristic have been isolated from substrates with high starch content [13,18,35]. Nevertheless, 6.6% of the LAB isolates could hydrolyze starch, and two of the LAB isolates selected (*L. mesenteroides* MAD3 and *E. mundtii* NAR1) showed amylolytic activity, even when they were isolated from fresh vegetables with low starch content. The enzyme is more efficiently expressed when the starch content of the medium is increased. Unban et al. [18] achieved improved efficiencies from LAB amylases at concentrations of up to 150 g of starch/L. Therefore, the effectiveness of starch hydrolysis in the presumptive LAB isolates reported here may be improved, since the test medium contained only 10 g/L of starch. Amylases are important enzymes used for the manufacture of fabric, food, medicines, and fermented products. Furthermore, LAB with high amylase activity might be useful for the production of lactic acid [18].

Even though the method employed for adhesion was initially reported for *S. aureus*, it has been successfully employed with LAB isolates [46,47]. More than half of the presumptive LAB isolates were positive for the adhesion test. The strains of *E. mundtii*, *L. mesenteroides*, and *E. faecium* identified produced exopolysaccharides. The production of exopolysaccharides by LAB is a feature that depends on the carbohydrate employed. However, strains of L. *mesenteroides* isolated from vegetables have been reported as exopolysaccharide producers from media supplemented with glucose [3,13,14] likewise, *E. mundtii* produced exopolysaccharides from glucose [13] whereas *Enterococcus* spp., did not [14]. Monika et al. [35] reported several strains of *E. faecalis* and *L. mesenteroides* isolated from traditional pickles in India that produced exopolysaccharides. Domingos-Lopez et al. [16] indicate that *Leuconostoc* strains mostly generate homopolysaccharides from glucose (glucans).

For the food industry, LAB strains that produce exopolysaccharides are of significant importance, since exopolysaccharides can enhance sensorial characteristics and stability [16]. Consequently, LABs are employed as starter cultures or coadjuvants for the manufacture of fermented products with high viscosity such as dairy products, puree and smoothies [3,13]. The production of exopolysaccharides is a desired feature for LAB employed as probiotic since adhesion and colonization of the gastrointestinal tract depend on their production [20].

## 5. Conclusions

LAB were isolated from fresh vegetables in Mexico and their antimicrobial and enzymatic activities were evaluated. The most critical strains showed antimicrobial activities possibly due to the production of antimicrobial peptides against Gram-positive and other LAB bacteria, yeast, and molds of importance for the food industry. They also exhibited enzymatic activities, such as protease, lipase, amylase, and citric acid metabolism and exopolysaccharide production. Potential technological applications of LAB isolated from fresh vegetables should be further evaluated due to the encouraging results. 

## Figures and Tables

**Table 1:** Indicator microorganisms used for the growth response assessment in Lactic Acid Bacteria (LAB) isolates determination of antimicrobial activity.

Indicator	ATCC	Incubation Conditions
*Bacillus cereus*	11,778	Aerobiosis, 37 °C, 24 h
*Listeria monocytogenes*	19,114	
*Staphylococcus aureus*	25,923	
*Escherichia coli*	35,218	
*Escherichia coli O157:H7*	43,888	
*Salmonella* Typhimurium	14,028	
*Lactococcus lactis*	Commercial	Anaerobiosis, 26 °C, 24 h
*Lacticaseibacillus casei* comb.nov.	Commercial	
*Aspergillus niger*	BUAP ^1^ collection	Aerobiosis, 26 °C, 48 h
*Penicillium expansum*	UDLAP ^2^ collection	
*Fusarium oxysporum*	UDLAP ^2^ collection	
*Candida albicans*	10,231	Aerobiosis, 26 °C, 24 h
*Saccharomyces cerevisiae*	9763	
*Candida tropicalis*	1369	

^1^ BUAP: Benemérita Universidad Autónoma de Puebla. ^2^ UDLAP: Universidad de las Américas Puebla.

**Table 2:** Fruit and vegetable sampled and the number of lactic acid bacteria isolated.

Sample	N° of Samples	N° Isolations/Shape	Sample	N° of Samples	N° Isolations/Shape
Red Apple	15	4 coccoid	Zucchini	15	2 rod, 2 coccoid
Green Apple	15	6 coccoid	Green Tomato	12	2 cocci, 2 coccoid
Pear	6	3 rod, 1 coccoid	Grape	3	1 rod
Pomegranate	3	2 cocci	Orange	6	1 coccus, 1 rod, 3 coccoid
Bell Pepper	6	3 coccoid	Peach	6	3 rod, 1 coccoid
Jalapeño Chili	18	1 coccus, 5 coccoid	Guava	15	2 cocci, 6 coccoid
Chilaca Chili	18	2 cocci, 3 rod, 5 coccoid	Corn	9	2 rod, 4 coccoid
Mandarin	3	coccoid	Lettuce	6	2 coccoid
Cucumber	12	2 coccoid	Nopal flower	3	1 coccoid
			Soybean	3	1 coccoid

**Table 3:** Number of strains of LAB isolated from fresh vegetable sources with enzymatic and adhesion capacity and percentage (In parenthesis).

Enzymatic Activity	Protease	Lipase	Citrate	Amylase	Adhesion
No activity	30 (39)	62 (81.6)	40 (52.6)	71 (93.4)	35 (46)
Medium	26(34)	11 (14.5)	13 (17)	3 (4)	17 (22)
High	20 (26)	3 (4)	23 (30)	2 (2.6)	24 (31.6)
Total positive	46 (60.5)	14 (18.4)	36 (47)	5 (6.6)	41 (54)
Total sample	76	76	76	76	76

**Table 4:** Adhesion activities of Lactic acid bacteria selected due to their multiple enzymatic activities showed.

SOURCE	CODE	ADHESION
Corn	ELO8	++
Bell pepper	PIM4	++
Cucumber	PEP12	++
Jalapeño	JAV15	+
Red apple	MR15	+
Tangerine	MAD3	+++
Orange	NAR1	+++
Guava	GUA13	++

**Table 5:** Molecular identification of LAB isolated from fresh vegetable sources selected according to their antimicrobial and enzymatic activities.

SOURCE	CODE	Selection Criteria	Pb ^1^	QUERY COVER %	IDENTITY %	RELATED STRAIN	ACCESSION #
Corn	ELO8	a, p, l, c, e	1011	100	100	*Enterococcus mundtii*	MN636717
Bell pepper	PIM5	a	1106	100	99.91	*Leuconostoc mesenteroides*	MN636704
Zucchini	CAL14	a	574	100	99.83	*Leuconostoc mesenteroides*	MN636705
Bell pepper	PIM4	a, p, l, c, e	897	99	99.11	*Enterococcus faecium*	MN636718
Jalapeño	JAV15	a, p, l, c	743	100	99.19	*Enterococcus mundtii*	MN636719
G. tomato	TOV9	a, l, c, e	1019	100	99.8	*Enterococcus mundtii*	MN636706
Cucumber	PEP11	a	780	100	95.27	*Enterococcus faecium*	MN636707
Cucumber	PEP12	a, p, l, c, e	991	100	99.6	*Leuconostoc mesenteroides*	MN636720
Red apple	MR15	p, l, m, c e	881	100	99.66	*Enterococcus mundtii*	MN636721
Tangerine	MAD3	p, l, m, c, e	1097	100	99.54	*Leuconostoc mesenteroides*	MN636708
Orange	NAR1	p, m, c, e	938	99	99.89	*Enterococcus mundtii*	MN636722
Guava	GUA13	p, l, c, e	708	99	99.43	*Leuconostoc mesenteroides*	MN636709

^1^ pb: Pair bases. a: Antimicrobial activity, p: proteolysis, l: lipolysis, m: amylase activity, c: citrate metabolism, e: exopolysaccharide production. #, number.
